# Biomarkers Obtained by Transcranial Magnetic Stimulation of the Motor Cortex in Epilepsy

**DOI:** 10.3389/fnint.2019.00057

**Published:** 2019-10-30

**Authors:** Melissa Tsuboyama, Harper Lee Kaye, Alexander Rotenberg

**Affiliations:** ^1^Neuromodulation Program, Department of Neurology, Division of Epilepsy and Clinical Neurophysiology, Boston Children’s Hospital, Boston, MA, United States; ^2^FM Kirby Neurobiology Center, Department of Neurology, Boston Children’s Hospital, Boston, MA, United States; ^3^Berenson-Allen Center for Noninvasive Brain Stimulation, Beth Israel Deaconess Medical Center, Boston, MA, United States

**Keywords:** biomarker (development), transcranial magnetic stimulation (TMS), epilepsy—abnormalities, classification, drug therapy, drug development and application, neuromodulation, motor cortex excitability

## Abstract

Epilepsy is associated with numerous neurodevelopmental disorders. Transcranial magnetic stimulation (TMS) of the motor cortex coupled with electromyography (EMG) enables biomarkers that provide measures of cortical excitation and inhibition that are particularly relevant to epilepsy and related disorders. The motor threshold (MT), cortical silent period (CSP), short interval intracortical inhibition (SICI), intracortical facilitation (ICF), and long interval intracortical inhibition (LICI) are among TMS-derived metrics that are modulated by antiepileptic drugs. TMS may have a practical role in optimization of antiepileptic medication regimens, as studies demonstrate dose-dependent relationships between TMS metrics and acute medication administration. A close association between seizure freedom and normalization of cortical excitability with long-term antiepileptic drug use highlights a plausible utility of TMS in measures of anti-epileptic drug efficacy. Finally, TMS-derived biomarkers distinguish patients with various epilepsies from healthy controls and thus may enable development of disorder-specific biomarkers and therapies both within and outside of the epilepsy realm.

## TMS Basics and Measures in Epilepsy

Epilepsy is among the most common neurologic disorders in childhood, and accompanies numerous neurodevelopmental disorders, particularly the autism spectrum disorders (ASDs; Levisohn, 2007; Tuchman and Rapin, 2002; Danielsson et al., 2005). For patient populations with epilepsy, biomarkers that reflect magnitudes of cortical excitation and inhibition are highly desirable as metrics of disease severity and target engagement by therapeutics. Transcranial magnetic stimulation (TMS) is a 30-year-old protocol for focal, noninvasive, electrical cortical stimulation that enables such measures across ages (Barker et al., 1985). In TMS, powerful fluctuating extracranial magnetic fields induce intracranial electrical current. When placed at the scalp, TMS induces electric current in the nearby cerebral cortex and allows for the operator to either measure or modulate focal cortical excitability.

The TMS “dose” per experiment is defined by hardware factors that affect the electromagnetic field. These include coil shape, size, electrical properties, and its placement relative to cortical structures. The stimulation parameter space also includes individual stimulus components such as pulse shape (rectangular, sinusoidal, exponential) and amplitude. The physiologic response to TMS is further determined by stimulus train parameters such as frequency, duration, inter-train interval, and the number of trains per unit time. The electric field generated by TMS is not measured *in vivo* but can be effectively modeled and represented in volts per meter (V/m). Alternatively, TMS-induced current density can be approximated in ampere per meter^2^ (A/m^2^; Peterchev et al., 2012).

**Figure 1:**
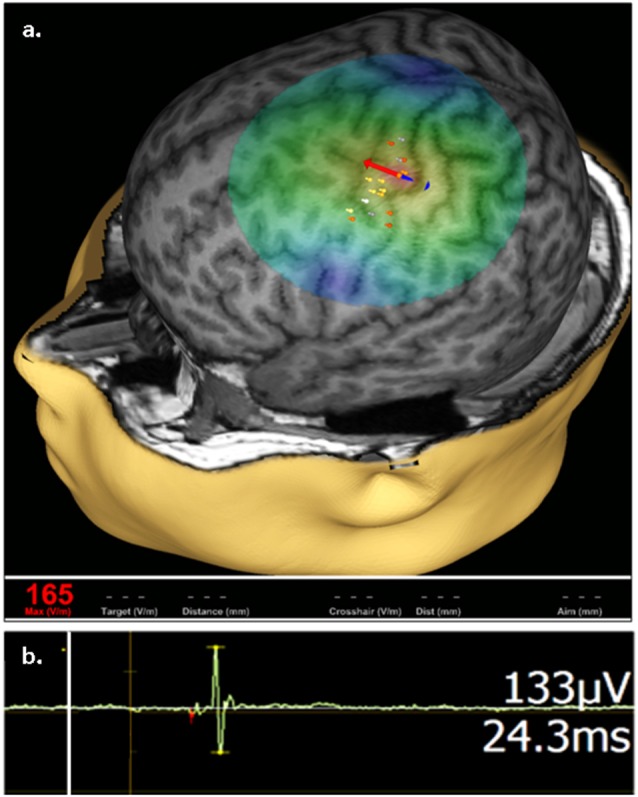
Representative transcranial magnetic stimulation (TMS) motor cortex activation. **(A)** An approximation of stimulating electric field (e-field) induced by a single TMS pulse is displayed on a 3D reconstruction of an individual’s anatomic magnetic resonance imaging (MRI), where field center is indicated by the junction between the red and blue arrows, indicating the direction of induced current, with corresponding e-field strength at the stimulation site (V/m) shown in red in the bottom left. The composite map of left hemispheric stimulation sites evoking motor evoked potentials (MEPs) of the right abductor pollicis brevis (APB) muscle, where intensity of response is color coded (heat map) from lowest (gray) to highest (white), are displayed on the cortical surface rendering. **(B)** Representative right APB MEP sample (green deflection) showing right APB resultant from left hemisphere stimulation, where the vertical line (white) corresponds to stimulus time. MEP amplitude and latency are indicated on the right.

Stimulation focality in TMS is in part governed by coil geometry. With a common type of TMS coil termed figure-of-eight, the volume of depolarized cortex with a single stimulus can be as small as 1 cm^3^. When positioned over the motor cortex, TMS by a figure-of-eight coil enables selective activation of intrinsic hand muscles in the limb contralateral to the stimulated hemisphere, without co-activation of more proximal muscle groups. Such motor cortex activation can be quantified with skin surface electromyography (EMG) that records a per-stimulus motor evoked potential (MEP) which predictably reflects the magnitude of stimulation and is the main outcome measure in TMS studies of the cortical excitation:inhibition (E:I) ratio (Figure 1; reviewed in Kobayashi and Pascual-Leone, 2003; Frye et al., 2008).

**Figure 2:**
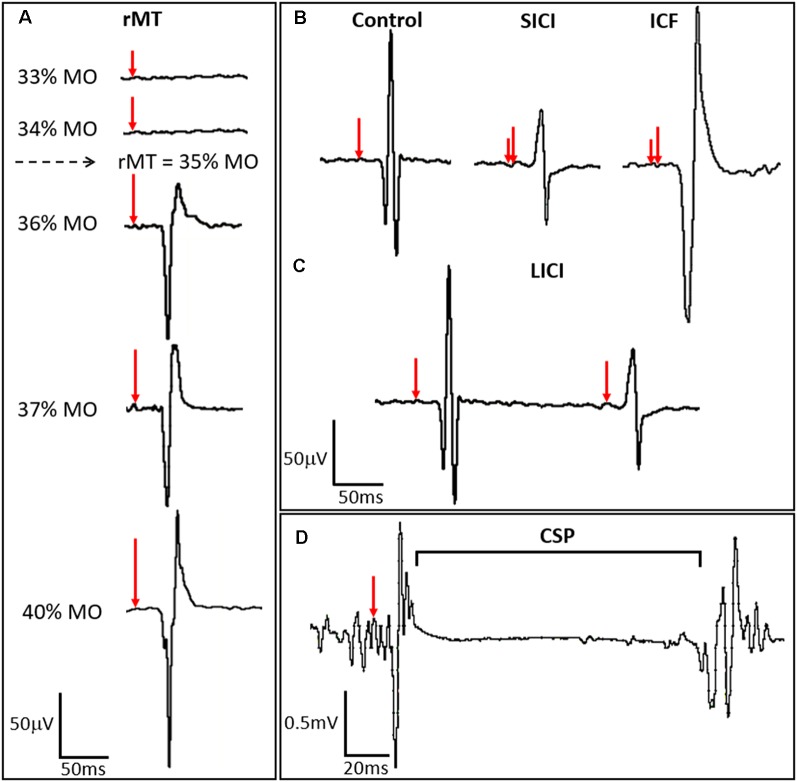
TMS-derived metrics of motor cortex excitation and inhibition. **(A)** Resting motor threshold (rMT) for the APB muscle is calculated by identifying the minimum stimulus strength, measured in percent machine output (% MO), that evokes an MEP of a fixed amplitude (typically ≥50 μV) in the APB at rest in a majority of trials. Stimulus strength is indicated in the left panel, with resulting MEPs shown in the right panel, where red arrows indicate the time of stimulation and percent stimulator output is proportionate to the arrow length. **(B)** ppTMS paradigms where a subthreshold conditioning stimulus (short red vertical line) followed by a supra-threshold test stimulus (longer red vertical line). At short inter-stimulus-intervals (ISIs) (1–5 ms) short interval intracortical inhibition (SICI) is seen with inhibition of the test MEP by the antecedent conditioning stimulus. At longer ISIs (10–20 ms), test MEP amplitude is enhanced relative to the control MEP, such that ICF is seen. **(C)** Still longer ISIs (50–300 ms) are applied with two suprathreshold stimuli in LICI protocols where the MEP resultant from the test stimulus is predictably lower in amplitude than the preceding MEP resulting from the conditioning stimulus. In **(D)** the cortical silent period (CSP), interruption of ongoing electromyography (EMG) activity for a voluntarily contracting target muscle, occurs following single-pulse TMS (spTMS).

TMS is unique among brain stimulation protocols in that it has both diagnostic and therapeutic potential. Three TMS protocols, all combined with surface EMG to measure MEP amplitude, are commonly employed to measure the cortical E:I ratio in epilepsy: (1) single-pulse TMS (spTMS); (2) paired-pulse TMS (ppTMS); and (3) repetitive TMS (rTMS). While there is appreciable device-to-device output variability of focality and magnitude of stimulation *via* TMS, the overall pulse width, and pulse shape (monophasic and biphasic) are relatively consistent across devices. Experimental devices with variable pulse width and shape are emerging, but thus to date are not widely implemented (Peterchev et al., 2011). In the most common embodiment of spTMS, the motor cortex is stimulated while muscle activation in a contralateral limb is monitored by surface EMG. spTMS, when used to determine the resting motor threshold (rMT), guides stimulation intensity in therapeutic rTMS (Figure 2A; Theodore, 2003; Ziemann, 2004).

spTMS coupled with surface EMG is also emerging as an important tool for functional topographic corticospinal tract mapping for purposes of presurgical planning (Lefaucheur and Picht, 2016; Hameed et al., 2017; Hannula and Ilmoniemi, 2017; Kaye et al., 2017b). ppTMS is an experimental technique, also delivered over the motor cortex, used to measure the cortical E:I ratio. In most common ppTMS protocols, two consecutive pulses are delivered to the hand motor region at a fixed inter-stimulus-interval (ISI) such that the MEP resultant from the second (test) stimulus is modulated by an antecedent (conditioning) stimulus. Depending on stimulus intensity and the ISI, ppTMS can reveal the magnitude of regional inhibitory or excitatory signaling strength (Figures 2B–D; Ziemann, 2003; Dhamne et al., 2015; Hsieh et al., 2017; Damar et al., 2018).

rTMS, delivered in trains lasting minutes, is most commonly used to modulate regional cortical excitability to suppress neuropsychiatric symptoms. In the motor cortex, rTMS is commonly administered in high-frequency (>10 Hz) or low-frequency (<1 Hz) protocols aimed to enhance or suppress, respectively the MEP amplitude to provide a metric of cortical plasticity. Notably, such suppression and facilitation varies among individuals (Maeda et al., 2002). The physiologic mechanisms by which rTMS modifies cortical excitability are not completely understood but resemble well-described phenomena of use dependent long-term potentiation (LTP) and long-term depression (LTD) of excitatory synaptic strength modulated by glutamatergic and gamma-aminobutyric acid (GABA)-ergic mediators (Fitzgerald et al., 2006; Pascual-Leone et al., 2011; Pilato et al., 2012; Muller et al., 2014; Yang et al., 2014).

Patterned rTMS protocols, such as theta burst stimulation (TBS) of the motor cortex, are also used to measure cortical plasticity, where the two principal patterns of TBS are continuous theta burst stimulation (cTBS) and intermittent theta burst stimulation (iTBS). Both consist of delivery of 50 Hz pulses in bursts of three with an inter-burst interval of 200 ms, which mimic endogenous theta rhythms. As with other TMS protocols intended to produce biomarkers, TBS relies on changes in MEP amplitude as the main outcome measure. cTBS paradigms involve continuous train of TBS over a given duration, which, in typically developing individuals, result in net MEP suppression or depression of corticospinal excitability in most instances. In iTBS, a 2-s train of TBS is repeatedly delivered every 10 s, —in healthy adults, this often (though not always) leads to MEP facilitation or corticospinal excitation (Jannati et al., 2017). Mechanistically, as with conventional rTMS, TBS protocols likely engage mechanisms of glutamatergic and GABA-ergic synaptic plasticity (Huang et al., 2005; Stagg et al., 2009; Oberman et al., 2011; Mix et al., 2015; Blumberger et al., 2018).

## Motor Cortex TMS Safety in Epilepsy

spTMS and ppTMS are well-tolerated by subjects at the extremes of age, with only rare and mild adverse events reported among infant, child and elderly populations (Liepert et al., 2001; Eyre, 2003; Hameed et al., 2017; Kaye et al., 2017a). Several TMS devices are now FDA-cleared for use in children and adults. TMS safety and tolerability in patients with epilepsy is underscored by the growing use of neuronavigated TMS (TMS is coupled with frameless stereotaxy; Figure 1) as a presurgical functional mapping tool in children with developmental delay and/or epilepsy who candidates for respective epilepsy surgery (Narayana et al., 2015; Kaye et al., 2017a,b).

Specifically among children, the subjective perception of TMS seems favorable. Children who undergo TMS generally rate the experience as positive with little adverse events occurring during the sessions. Some children have even reported TMS to be more enjoyable than watching TV or going to the dentist (Garvey et al., 2001). Notably, in patients with epilepsy, per-subject risk for seizure with rTMS, spTMS or ppTMS is higher, yet is less than 3% crude per-subject risk (Schrader et al., 2004; Bae et al., 2007; Rossi et al., 2009; Pereira et al., 2016). The favorable safety profile of TMS has allowed for its use for studying cortical excitability in elderly patients with neurodegenerative disorders (Liepert et al., 2001).

TMS protocols are also available in rats, which underscores the versatility of motor cortex TMS as a protocol that is available in both clinical and preclinical arenas (Rotenberg et al., 2008; Hsieh et al., 2011; Gersner et al., 2015; Tang et al., 2016; Damar et al., 2018; Hameed et al., 2018).

## TMS-Derived Measures of Cortical Excitability, and Their Modulation by Antiepileptic Drugs

A range of cortical excitability measures that are affected by both epilepsy and antiepileptic drugs (AEDs) can be obtained by TMS coupled with surface EMG. Motor threshold (MT) is often defined as the minimum percentage of stimulator output (% MO) that evokes an MEP of a fixed amplitude (typically >50 μV) in a target muscle either at rest (rMT) or during voluntary contraction (active motor threshold, aMT) in a majority of trials (Theodore, 2003; Ziemann et al., 2015; Figure 2A). The cortical silent period (CSP) is a TMS-induced interruption of activity in the EMG of the voluntarily contracting target muscle. The early segment of the CSP is related to spinal inhibition while the later segment is hypothesized to be of motor cortical origin. Short-interval intracortical inhibition (SICI) results from inhibition of the test MEP by a conditioning stimulus. This ppTMS protocol involves the application of a subthreshold conditioning stimulus and supra-threshold test stimulus at short ISIs (1–5 ms). Stimulation using a similar protocol but with longer ISIs of 10–20 ms results in intracortical facilitation (ICF; Kobayashi and Pascual-Leone, 2003; Ziemann, 2004). Long-interval intracortical inhibition (LICI) is measured using ppTMS with two supra-threshold stimuli applied at long ISIs of 50–300 ms in which the conditioning stimulus inhibits the test MEP. Such TMS-EMG parameters are summarized in Table 1 (Rotenberg, 2018) and illustrated in Figures 2B–D.

**Table 1:** Transcranial magnetic stimulation-electromyography (TMS-EMG) metrics.

TMS-EMG parameter	Protocol*	Likely mechanism	Examples of change with medication
**Resting motor threshold (rMT)**	Single-pulse TMS: measure of stimulus strength necessary for a motor response (recorded either by visual inspection or EMG)	Cortical motor neuron voltage-gated sodium channel-mediated membrane excitability	Increased by voltage-gated sodium channel blockers (e.g., phenytoin, lacosamide) and voltage-gated potassium channel openers (e.g., retigabine)
**Cortical silent period (CSP)**	Single-pulse TMS: measure of pause in voluntary EMG activity after TMS	GABA_B_-mediated and GABA_A_-mediated motor cortex inhibition	Increased by GABA_B_ agonists (e.g., baclofen); increased by GABA_A_ positive allosteric modulators (e.g., lorazepam)
**Short-interval intracortical inhibition (SICI)**	Paired-pulse TMS: subthreshold conditioning stimulus precedes test stimulus by 1–5 ms	GABA_A_-mediated regional cortical inhibition	Increased by GABA_A_ positive allosteric modulators (e.g., lorazepam)
**Intracortical facilitation (ICF)**	Paired-pulse TMS: subthreshold conditioning stimulus precedes test stimulus by 10–20 ms	Glutamate (NMDA and AMAPA receptor types)- mediated excitation	Decreased by NMDA-type and AMPA-type glutamate receptor antagonists (e.g., memantine)
**Long-interval intracortical inhibition (LICI)**	Paired-pulse TMS: suprathreshold conditioning stimulus precedes test stimulus by 50–300 ms	GABA_B_-mediated inhibition and (likely) GABA_A_-mediated network inhibition	Increased by GABA_B_ agonists (e.g., baclofen); increased by GABA_A_ positive allosteric modulator (e.g., pentobarbital)

*Protocols vary slightly among laboratories; nearly always obtained from intrinsic hand muscles.

rMT reflects the degree of cortical excitability which is affected by voltage-gated sodium channel blockers. Carbamazepine (CBZ), lacosamide (LCM), lamotrigine (LTG), and phenytoin (PHT), increase rMT compared to the rMT in drug-naïve patients with epilepsy and in patients without epilepsy; these changes are reversible with withdrawal of the given medication (Chen et al., 1997; Manganotti et al., 1999; Kimiskidis et al., 2005; Lee et al., 2005; Li et al., 2009; Lang et al., 2013; Ziemann et al., 2015). The effect of levetiracetam (LEV) on rMT remains uncertain, as Sohn et al. (2001) show no change in rMT with LEV administration, while Reis et al. (2004) report a significant increase in rMT in patients taking LEV (Sohn et al., 2001; Reis et al., 2004).

Notably, there is a dose-dependent relationship between rMT and certain AEDs. Lee et al. (2005) measured serial rMT and serum drug levels in healthy volunteers taking gradually increasing dosages of CBZ over 5 weeks followed by an abrupt cessation of the drug. rMT increased with increasing serum drug levels of total and free CBZ (Lee et al., 2005). In 7 of 10 patients, upon abrupt CBZ cessation, rMT remained elevated initially and then gradually returned to the baseline over several days despite the abrupt drop in serum CBZ levels. The sustained increase in rMT despite absent serum CBZ level indicates that the rMT (and perhaps other TMS-derived E:I metrics) may distinguish between drug pharmacodynamics and pharmacokinetics (Lee et al., 2005). As with CBZ, Lang et al. (2013) show a trend towards a dose-responsive effect on rMT with LCM dosages of 200 mg and 400 mg (Lang et al., 2013).

The effect a drug has on rMT can also provide information regarding its antiepileptic mechanism of action. For example, valproate (VPA) has no significant effect on rMT in healthy volunteers. VPA does increase rMT in focal epilepsies while its effect on rMT in patients with idiopathic generalized epilepsy (IGE) remains unclear as there are contradictory findings among studies (Reutens et al., 1993; Kazis et al., 2006; Li et al., 2009; Zunhammer et al., 2011; Badawy et al., 2014). Topiramate (TPM), like VPA has several mechanisms of action, including voltage gated sodium channel antagonism, but does not affect rMT while reducing ICF as its anti-epileptic properties stem primarily from inhibition of ligand-gated AMPA subtype glutamate receptors and agonist effects on some subtypes of the GABA_A_ receptor (Angehagen et al., 2005). If VPA and TPM do not modulate the rMT, then while *in vitro* they may indeed have sodium channel blocking properties, these are not prominent *in vivo*, or in humans—thus the antiepileptic efficacy of these AEDs is less likely due to the sodium channel properties.

Antiepileptic medications also affect CSP duration, SICI, and LICI—all measures of components of GABA-ergic inhibition. CSP duration and SICI reflect motor cortical postsynaptic inhibition. ICF reflects glutamate receptor-mediated excitability that counters the inhibitory circuits reflected in SICI. GABA_A_ receptor positive allosteric modulators such as benzodiazepines prolong short CSPs when low-intensity stimulation is used and shorten long CSPs when high-intensity stimulation is used. SICI is thought to represent fast inhibitory postsynaptic potentials (IPSPs) in corticospinal neurons mediated by α2- or α3-GABA_A_ receptors. SICI is predictably enhanced by benzodiazepines and barbiturates. LICI reflects slow IPSPs mediated in part by GABA_B_ receptors. LICI, where the long interval between pulses enables signals to propagate across multiple local and distal networks also likely reflects an aggregate inhibitory tone that is mediated by the GABA_A_ receptor system (Hsieh et al., 2011). As expected, vigabatrin increases LICI, while there are conflicting reports on lorazepam’s effect on LICI (Ziemann et al., 1996; Teo et al., 2009). In animal models, LICI is also enhanced by pentobarbital and suppressed by the GABA_A_ receptor blocker pentylenetetrazole (PTZ; Hsieh et al., 2011).

Notably, however, tiagabine has a more complex interaction between the GABA_A_ and GABA_B_ receptor subtypes. Increased extracellular GABA availability in this instance results in predictable CSP prolongation and increased LICI. However, tiagabine decreases SICI which is controlled by presynaptic GABA_B_ receptor-mediated autoinhibition of inhibitory interneurons. This contributes to the net increase in excitatory response as illustrated by the increase in ICF with tiagabine administration.

N-methyl-D-aspartate (NMDA)-receptor antagonists such as dextrorphan, the active metabolite of the prodrug dextromethorphan, and memantine, and benzodiazepines such as diazepam decrease ICF while enhancing SICI (Schwenkreis et al., 1999). Table 2 summarizes the effects of various classes of drugs on these variables.

**Table 2:** Antiepileptic drug effect on TMS parameters.

Class/Drug	MT	CSP	SICI	ICF	LICI	Dose-responsive?	Comments
**Sodium channel**
Carbamazepine	↑	-	-	-		Yes	
Lacosamide	↑	-	-	-		Yes	
Lamotrigine	↑	-	-	-		Yes	
Oxcarbazepine	↑	-	-	-			
Phenytoin	↑	-	-	-			
**Potassium channel**
Retigabine	↑		-	-	-		
XEN1101	↑					Yes	
**GABA_A_ receptor**
Diazepam	-	↓^§^	↑	↓	-		
Lorazepam	-	↑^§^	↑	↓,-	↓		Conflicting reports on effects on ICF in healthy controls and a subject with spinal cord stimulator
Tiagabine	-	↑	↓	↑	↑		
Vigabatrin	-	↑	-	↓	↑		
**Calcium channel**
Gabapentin^†^	-	↑	↑	↓		No	
Pregabalin^†^	-	↑	↓	-	↑	No	
**NMDA glutamate receptor**
Dextromethorphan	-	-	↑	↓			
Memantine	-	-	↑	↓			
**AMPA glutamate receptor**
Perampanel	↑						
Topiramate^†^	-	-	↑	↓/-**		Yes	Dose-responsive relationship with SICI
**Other**
Levetiracetam	↑, -	-	-	-			Conflicting reports on effects on MT in healthy controls
Valproic acid^†^	↑,-	-	-	-			Increased MT in IGE, no change in healthy controls

MT, motor threshold; CSP, cortical silent period; SICI, short-interval intracortical inhibition; ICF, intracortical facilitation; LICI, long-interval intracortical facilitation; IGEm, idiopathic generalized epilepsy; ^§^stimulation intensity-dependent; ^**^trending towards decrease but not statistically significant; ^†^multiple mechanisms of action; ↑: increase; ↓: decrease; -: no change; blank cells, not tested; ,: conflicting results.

## TMS-EMG Measures in Epilepsy Pharmacotherapy

While changes in TMS parameters following acute drug administration aid in the identification of mechanisms of action of various drugs (or identify the mechanism of TMS-derived phenomena) at the receptor level, the effect of long-term administration of antiepileptic medications on these parameters may serve as a proxy for prognosticating efficacy of antiepileptic medications. A longitudinal study with 1-year follow-up illustrated a reduction in cortical excitability in patients with IGE or focal epilepsy who became seizure-free with anti-seizure medications (Badawy et al., 2010). In fact, while the rMTs were overall higher in these patients than in the control subjects without epilepsy, only the subset of patients with epilepsy who became seizure-free demonstrated an increase in rMT. These findings were independent of seizure type, seizure frequency, patient current age or age at seizure onset, and serum levels of the medication.

A subsequent study with a 3-year follow-up period compared measures of cortical inhibition and facilitation in patients with IGE or focal epilepsy, between those who remained refractory to antiepileptic drugs and those who achieved seizure freedom (Badawy et al., 2013). The mean rMT was higher in the affected hemisphere in patients with focal epilepsy compared to the unaffected hemisphere prior to initiation of anti-seizure medications. There was no difference in pre-drug treatment rMT between control patients without epilepsy and patients with IGE. Patients whose focal seizures remained refractory following initiation of one AED had an increase in rMT in the contralateral (unaffected) hemisphere such that there was no difference between the rMT in the two hemispheres. In patients who achieved seizure freedom on monotherapy, mean rMTs were higher in bilateral hemispheres in patients with IGE and patients with focal epilepsy compared to those patients who remained refractory. This pattern was maintained by the 30–36 months follow-up time-frame. Patients with refractory focal epilepsy developed a hyperexcitable contralateral hemisphere (at ISIs of 2 and 5 ms) at 30–36 months. A similar hyperexcitable response was also seen during a time of continued seizures in patients who would become seizure-free after the second medication. When those patients became seizure-free, however, there was subsequent normalization of all ISIs by the 30–36 months time frame. For the seizure-free patients in this cohort, rMT was higher than that measured in non-epilepsy controls, and SICI and LICI gradually increased to normal or near normal-values at most ISIs (Badawy et al., 2013).

These results suggest a close association between seizure freedom and normalization of TMS-derived cortical excitability metrics with prolonged AED use in patients with both focal and generalized epilepsy. Whether this effect is due to a change within the brain’s predisposition to generate seizures or attributable to the cessation of continued seizure activity is unknown. However, regardless of the drug(s) used, a common effect of successful AED treatment is the restoration of normal responses to TMS.

## TMS-EMG Measures in Nonpharmacologic Treatment of Epilepsy

As SICI reflects the activity of intracortical inhibitory circuits, particularly that of GABA_A_ receptor-mediated activity (Ziemann, 2004), serial SICI measurements over a given time course provide an index for GABA-mediated motor cortex inhibition (Maeda et al., 2002). Cantello et al. (2007) tested a range of TMS-derived metrics in healthy volunteers placed on the ketogenic diet (KD), to find that short-term KD (14-days) was followed by significant SICI enhancement. Notably, rMT was unchanged after KD initiation suggesting a prominent GABA_A_ receptor contribution to the KD antiepileptic mechanism of action (Cantello et al., 2007).

Di Lazzaro et al. (2004) compared baseline TMS measures (rMT, SICI) for five patients with medically-refractory epilepsy who underwent vagus nerve stimulator (VNS) implantation. TMS measures were obtained in the stimulator-off and stimulator-on conditions. Patient rMT was higher than healthy age-matched controls but did not change with the VNS on. In contrast, SICI significantly increased when the VNS was on. As with KD, these results indicate a TMS-derived marker of target engagement, and a capacity for TMS-EMG to identify a GABAergic contributor to an antiepileptic intervention’s mechanism (Di Lazzaro et al., 2004).

## TMS in The Anti-Epileptic Drug Development Pipeline

Changes in cortical excitability detected by ppTMS can be used in both preclinical and clinical studies to develop and assess the efficacy of novel AEDs. Huperzine A (HupA), a traditional Chinese medicine administered for treatment of epilepsy, is a naturally occurring esquiterpene alkaloid compound found in the firmoss *Huperzia serrata* that is both an acetylcholinesterase inhibitor and N-methyl-D-aspartate receptor (NMDAR) antagonist. Preclinical trials show that HupA suppresses seizures in a range of rodent epilepsy models. By ppTMS and differential pharmacology, Gersner et al. (2015) identified a potent GABAergic effect of HupA that was reflected in preserved paired-pulse inhibition of the MEP when rats were co-administered HupA and PTZ (a convulsant and GABA-A receptor blocker), and augmented LICI when HupA was administered in isolation (Gersner et al., 2015). The group concluded that at least in part the anti-seizure HupA effects may result from the enhancement of cortical GABAergic tone. These initial preclinical results justify continued preclinical and clinical investigations of HupA as a potential new anti-seizure drug compound.

Retigabine (RTG) is a newer generation drug that acts as a positive allosteric opener of KCNQ2–5 potassium channels to increase potassium efflux resulting in neuronal hyperpolarization and a decrease in neuronal excitability. In a cross-over, double-blind, placebo-controlled, randomized control trial, Ossemann et al. (2016) used single-pulse TMS with a figure-of-eight coil to measure rMT and aMT, and intensity to obtain a 1 mV peak-to-peak amplitude potential (SI1mV), and ppTMS to measure SICI, LICI, and ICF (Ossemann et al., 2016). Baseline measurements and measurements 2 h following administration of an oral dose of 400 mg RTG or placebo were obtained. RTG increased rMT, aMT, and S1mV compared to placebo, suggesting that RTG decreases neuronal excitability by increasing the resting potential as hypothesized from *in vitro* studies. However, SICI/ICF, and LICI were not significantly different between the RTG and placebo groups, suggesting that RTG does not affect intracortical inhibition (Ossemann et al., 2016).

XEN1101 is a voltage-gated potassium channel opener in the early stages of development that has shown promising preliminary data as a new antiseizure drug through the use of TMS. In a Phase 1 open-label study, spTMS was used to measure rMT in healthy control subjects taking 10 mg, 15 mg, or 20 mg of XEN1101. Premoli et al. (2019) found that 20 mg of XEN1101 decreased cortical excitability compared to the lower dosages (Premoli et al., 2019). In a subsequent double-blind, randomized, two-period crossover study, XEN1101 elevated rMT in a plasma concentration-dependent fashion. These encouraging findings support that XEN1101 reduces corticospinal and cortical excitability in a plasma concentration-dependent manner and have prompted plans for Phase 2 clinical trials.

## TMS-Derived Metrics in Rare Epilepsies

As expected, alterations in cortical inhibitory networks are also seen in various genetic and metabolic epilepsies (Table 3). SICI is decreased in patients with progressive myoclonic epilepsies (PME), including Unverricht-Lundborg disease (ULD), Lafora body disease (LBD), progressive myoclonic ataxia, sialidosis, and myoclonic epilepsy with ragged red fibers (MERRF). In patients with noncortical myoclonus, such as those with DYT-1 myoclonus-dystonia syndrome, SICI can be normal or mildly impaired. These findings help elucidate the pathophysiology of these diseases. For example, mutations in laforin or malin lead to formation and accumulation of neuronatin aggregates, typically found in parvalbumin-positive inhibitory interneurons (PVINs), resulting in significant reduction in and degeneration of PVINs on brain biopsy of patients with LBD. This reduction in cortical inhibition is reflected by the decreased SICI, and simultaneously illustrates the role of cortical PVINs on SICI (Rotenberg, 2018).

**Table 3:** TMS-EMG metrics in rare epilepsies.

Subjects	Findings (relative to control); Comments				
	MT	SICI	ICF	LICI	CSP
PME	-/↑ (rMT unchanged or increased; aMT increased in ULD and LBD)	↓	↓ (LBD only)	↓ (- in ULD)	-
DS	-	↓	-	-
SSADHD	↑/- (unchanged after taurine treatment)	-/↑ (increased after taurine treatment)	↓/-(unchanged after taurine treatment)	↑/-	↓
LGS	↑	↑	↑	↑	

*MT, motor threshold; SICI, short-interval intracortical inhibition; ICF, intracortical facilitation; LICI, long-interval intracortical facilitation; CSP, cortical silent period; PME, primary myoclonus epilepsies; LBD, Lafora body disease; ULD, Unverricht-Lundborg disease; DS, Dravet syndrome; SSADHD, succinic semialdehyde dehydrogenase deficiency; LGS, Lennox–Gastaut Syndrome; ↑, increase; ↓, decrease; -: no change; blank cells, not tested; results for cohort 1, results for cohort 2; /: conflicting results*.

SICI is also reduced in SCN1A-related epilepsies such as Dravet syndrome (DS), which again reflects abnormal cortical inhibition networks, while the other TMS-derived markers of cortical excitability remain normal (Stern et al., 2017). These findings are consistent with preclinical data showing PVIN and somatostain-positive inhibitory interneuron dysfunction in murine DS models (Tai et al., 2014).

LICI abnormalities can also indicate cortical inhibitory network dysfunction, but, unlike SICI, reflect GABA_B_ receptor activity. In patients with succinic semialdehyde dehydrogenase (SSADH) deficiency, LICI is reduced and CSP is shortened while SICI is preserved. These findings are supported by preclinical data showing GABA_B_ receptor loss and/or dysfunction in a murine SSADH deficiency model (Rotenberg, 2018).

Additionally, rMT is increased in young patients with SSADH deficiency compared to their parents who are heterozygous for the causal pathogenic variant. However, this may be related to the age-dependent changes in rMT seen in healthy children and in patients with epilepsy (Hameed et al., 2017; Säisänen et al., 2018). Increase in rMT can also be found in several forms of cortical myoclonus, such as PME. In contrast to patients with chronic refractory IGE or those with chronic refractory FE, interictal cortical excitability is decreased in Lennox–Gastaut syndrome (LGS), where cortical excitability was lower in LGS patients. Cortical excitability was also lower in LGS when compared with healthy controls. This low cortical excitability across TMS measures thus distinguishes LGS from other medically refractory epilepsy syndromes (often showing measures of increased cortical excitability; Badawy et al., 2012).

## Conclusion

Noninvasive stimulation of the motor cortex with TMS has practical and easily attainable implications for identification of biomarkers in epilepsy. TMS-derived metrics of E:I properties resultant from motor cortex stimulation paradigms elucidate mechanisms of action, pharmacodynamics, and pharmacokinetics of AEDs, and speak to the underlying pathophysiology of a range of epilepsy disorders. A range of established protocols and metrics are available in numerous laboratories, and can not only be deployed to measure disease severity, predict and measure response to existing treatments in epilepsy, but also aid in the identification and development of novel areas for target engagement in the treatment of an array of disorders.

## Author Contributions

All authors wrote and revised the manuscript, approved the final version, and agreed to be accountable for the content of the work.

## Conflict of Interest

The authors declare that the research was conducted in the absence of any commercial or financial relationships that could be construed as a potential conflict of interest.

## Abbreviations

TMS: transcranial magnetic stimulation
V/m: volts per meter
A/m2: ampere per meter2
T: tesla
spTMS: single-pulse TMS
ppTMS: paired-pulse TMS
rTMS: repetitive TMS
EMG: electromyography
rMT: resting motor threshold
E:I: excitation to inhibition ratio
APB: abductor pollicis brevis
ISI: inter-stimulus-interval
LTP: long-term potentiation
LTD: long-term depression
TBS: theta burst stimulation
cTBS: continuous theta burst stimulation
iTBS: intermittent theta burst stimulation
MEP: motor evoked potential
CS: corticospinal
AEDs: antiepileptic drugs
MT: motor threshold
% MO: percent machine output
aMT: active motor threshold
CSP: cortical silent period
LICI: long-interval intracortical inhibition
SICI: short-interval intracortical inhibition
CBZ: carbamazepine
LCM: lacosamide
LTG: lamotrigine
PHT: phenytoin
LEV: levetiracetam
VPA: valproate
IGE: idiopathic generalized epilepsy
TPM: topiramate
AMPA: alpha-amino-3-hydroxy-5-methy;-4-isoxazole propionic acid
KD: ketogenic diet
VNS: vagus nerve stimulator
HupA: Huperzine A
NMDAR: N-methyl-D-aspartate receptor
PTZ: pentylenetetrazole
RTG: Retigabine
PME: progressive myoclonic epilepsies
ULD: including Unverricht-Lundborg disease
LBD: Lafora body disease
MERRF: myoclonic epilepsy with ragged red fibers
PVINs: parvalbumin-positive inhibitory interneurons
DS: Dravet syndrome
(SSADH) deficiency: succinic semialdehyde dehydrogenase.
